# Post-embryonic endogenous expression and localization of LET-60/Ras in *C. elegans*

**DOI:** 10.17912/micropub.biology.000931

**Published:** 2023-08-25

**Authors:** Ranjay Jayadev, Qiuyi Chi, David R Sherwood

**Affiliations:** 1 Department of Biology, Duke University, Durham, NC, USA

## Abstract

Ras GTPases regulate many developmental and physiological processes and mutations in Ras are associated with numerous human cancers. Here, we report the function, levels, and localization of an N-terminal knock-in of mNeonGreen (mNG) into *C. elegans* LET-60 /Ras. mNG:: LET-60 interferes with some but not all LET-60 /Ras functions. mNG:: LET-60 is broadly present in tissues, found at different levels in cells, and concentrates in distinct subcellular compartments, including the nucleolus, nucleus, intracellular region, and plasma membrane. These results suggest that mNG:: LET-60 can be a useful tool for determining LET-60 levels and localization once its functionality in a developmental or physiological process is established.

**Figure 1. mNeonGreen (mNG) knock-in site, phenotypes, and localization of mNG:: LET-60 /Ras:**
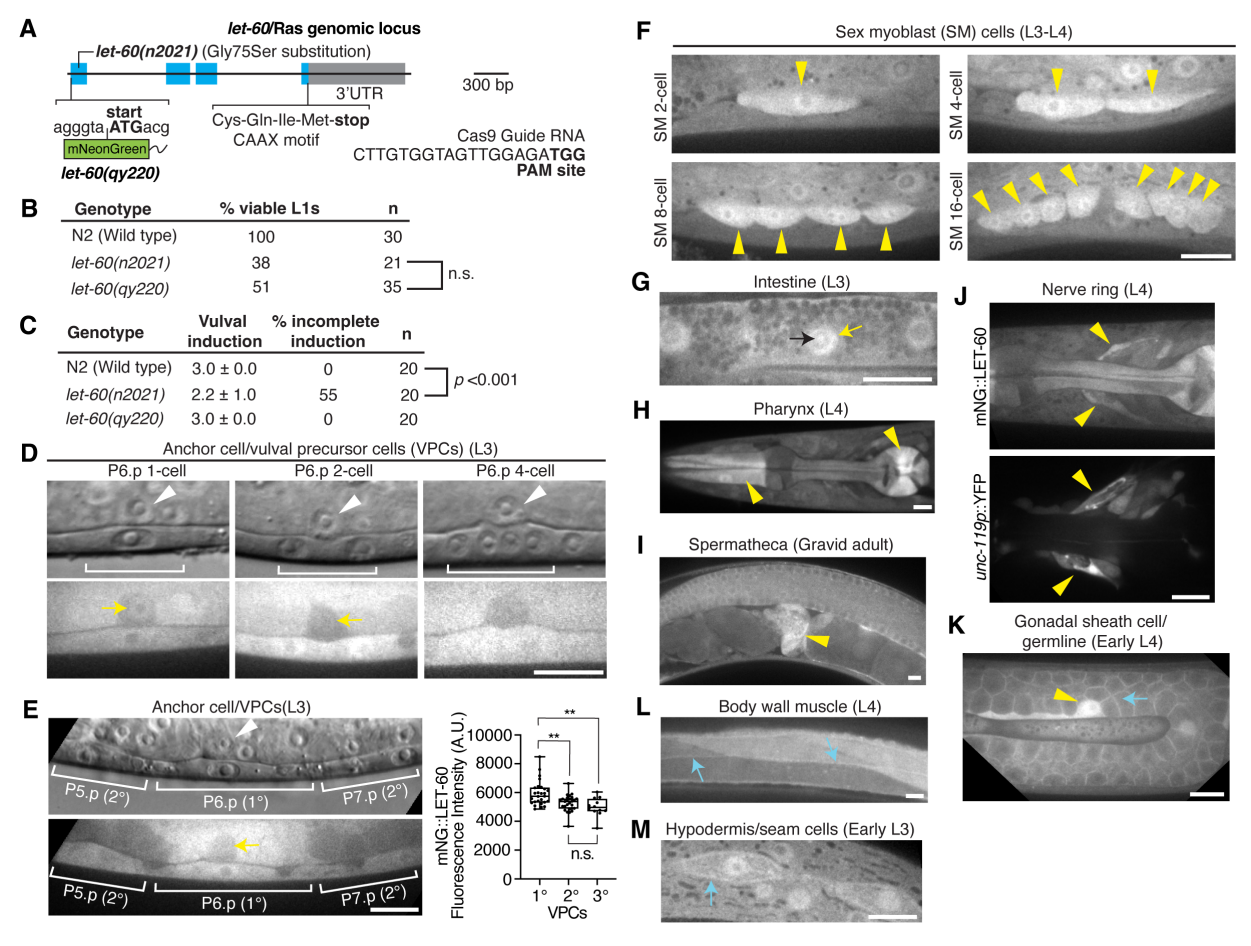
**(A)** Exon-intron gene structure of *let-60* /Ras. Exons are shown in blue and 3’UTR is indicated in grey. The position of the endogenous mNG tag and Cas9 guide RNA sequence used to generate the mNG-tagged *let-60 ( qy220 )* allele are highlighted. The *let-60 ( n2021 )* reduction-of-function allele harbors a point mutation in the first exon that causes the substitution of glycine to serine at codon 75. The C-terminus of LET-60 contains a CAAX motif that is the site of post-translational addition of a farnesyl isoprenoid. **(B)** Viability of L1 larvae with the genotypes listed. The percentage indicates the proportion of living L2 animals. n.s. (not significant), *p* > 0.05; Fisher’s exact test. **(C)** Assessment of vulval induction at the L3 larval stage in the listed genotypes (left). The average number of vulval precursor cells (VPCs) that adopt vulval fates in animals examined (middle) and percentage of animals with incomplete vulval induction (right) are shown. The *p* value was calculated with a Fisher’s exact test. **(D)** Fluorescence images of mNG:: LET-60 localization during vulval induction. The corresponding brightfield images indicate the uterine anchor cell (white arrowheads) and the central vulval precursor cell P6.p and its descendants (brackets). Yellow arrows indicate nuclear mNG:: LET-60 signal within the anchor cell. **(E)** Left, mNG:: LET-60 localization in VPCs. The white arrowhead and yellow arrow denote the anchor cell in the brightfield and fluorescence images, respectively. Right, boxplot of mean mNG:: LET-60 fluorescence intensity in the 1°, 2°, and 3° VPCs (n > 12 for each group). ** *p* < 0.01, n.s. (not significant), *p* > 0.05; Kruskal-Wallis *H* test with post hoc Dunn’s test. Box edges represent the 25th and 75th percentiles, the line in the box denotes the median value, and whiskers mark the minimum and maximum values. A.U. (arbitrary units). **(F)** Intracellular and nuclear enrichment of mNG:: LET-60 in sex myoblast cells (yellow arrowheads). **(G)** Prominent mNG:: LET-60 localization to the nucleus in intestinal cells at the L3 larval stage (yellow arrow, n = 20/20 animals examined). Note that nucleolar mNG:: LET-60 signal was observed in some intestinal cells (black arrow, n = 5/20 animals examined). **(H)** Intracellular enrichment of mNG:: LET-60 at the anterior and posterior regions of the pharynx (arrowheads). **(I)** mNG:: LET-60 is present at high levels intracellularly in the adult spermatheca (arrowhead). **(J)** Top, prominent localization of mNG:: LET-60 in neurons associated with the nerve ring (arrowheads). Bottom, nerve ring region visualized with the pan-neuronal marker *unc-119p* ::YFP (Calixto et al., 2010). **(K)** mNG:: LET-60 localizes prominently to the plasma membranes of germ cells (cyan arrow) and is abundant in the gonadal sheath cell (yellow arrowhead). **(L)** mNG:: LET-60 localizes to the body wall muscle cell membranes (arrows). **(M)** mNG:: LET-60 localizes to seam cell membranes in larvae (arrows). All fluorescence images shown are representative of the respective developmental stages and tissues (n > 10 animals examined for each stage). Scale bars, 10 µm.

## Description

Gain-of-function mutations in human Ras genes are found in ~20% of cancers, and there is intense interest in understanding the regulation, function, and therapeutic targeting of RAS (Fernandez-Medarde et al., 2021; Hobbs et al., 2016; Prior et al., 2020). The monomeric small GTPase Ras is highly conserved in metazoans and is a key component in many signaling pathways that regulate diverse normal cellular processes (Fernandez-Medarde et al., 2021). Genetic studies of the *C. elegans* Ras ortholog *let-60* gene have significantly advanced our understanding of Ras regulation and function in vivo (Rasmussen & Reiner, 2021; Sundaram, 2013) . The LET-60 /Ras protein regulates many aspects of development and homeostasis, such as excretory duct cell fate and differentiation, germline development, vulval cell fate, sex myoblast migration, muscle differentiation, tissue connection, axon outgrowth, exopher-mediated neuronal extrusion, olfaction, and learning and memory (Abdus-Saboor et al., 2011; Beitel et al., 1990; Bulow et al., 2004; Cooper et al., 2021; Gyurko et al., 2015; Han et al., 1990; Hirotsu et al., 2000; Park et al., 2023; Sundaram et al., 1996; Sundaram, 2013; Vaid et al., 2013) . Consistent with its many functions, a *let-60* transcriptional reporter is expressed broadly during *C. elegans* larval development (Dent & Han, 1998) . The localization of the LET-60 /Ras protein in *C. elegans* , however, is unknown.

CRISPR-Cas9 mediated fluorescent protein knock-in is a powerful approach for determining endogenous protein levels and localization in *C. elegans* and other model organisms (Dickinson et al., 2015; Keeley et al., 2020; Levic et al., 2021) . Yet, there are no reported fluorescent protein knock-ins for any Ras orthologs and only localization of transgene-driven GFP-tagged Ras proteins have been reported in yeast and zebrafish (Manandhar et al., 2010; Onken et al., 2006; Santoriello et al., 2010) . One potential challenge is that Ras proteins are small and contain highly conserved domains responsible for binding to and hydrolysis of guanine nucleotides. Ras proteins also functionally interact with GTPase activating proteins (GAPs), guanine nucleotide exchange factors (GEFs), and effectors (Hobbs et al., 2016) . As a result, fusion of a fluorescent protein within the central region of Ras proteins would likely interfere with Ras function. In addition, the C-terminus of Ras proteins harbor a CAAX domain that acts as a signal for the post-translational addition of a farnesyl isoprenoid (Fernandez-Medarde et al., 2021). Prenylation involves isoprenoid lipid attachment to the cysteine of the CAAX sequence, followed by proteolysis of the -AAX sequence, and then carboxyl methylation (Gao et al., 2009) . This promotes anchoring of Ras to membranes (Michaelson et al., 2005) . The proteolytic cleavage following lipid attachment, however, precludes fusing fluorescent proteins to the C-terminus as the fluorophore would either be removed or possibly interfere with prenylation and protein function. This leaves the N-terminus of Ras as the most promising site for fluorescent protein tagging and is the location of GFP tags in yeast and zebrafish Ras proteins (Manandhar et al., 2010; Onken et al., 2006; Santoriello et al., 2010) .

To determine the expression and localization of LET-60 /Ras we used CRISPR-Cas9 mediated genome editing to fuse mNeonGreen (mNG) to the N-terminus of the *let-60* gene ( *let-60 ( qy220 ))* ( **Figure 1A** and **Methods** ). We examined two functions of the LET-60 protein to determine the functionality of mNG:: LET-60 . First, we determined L1 and L2 stage viability, as LET-60 function is required for excretory duct cell fate and differentiation and perturbations in the duct cell lead to early larval death (Abdus-Saboor et al., 2011; Han et al., 1990) . Approximately ~50% of mNG:: LET-60 worms died in the L1 stage, which is similar to animals harboring the weak loss-of-function allele *let-60 ( n2021 )* ( **Figure 1B** ) (Gyurko et al., 2015). Next, we examined the role of LET-60 in vulval induction, where EGF signaling from the uterine anchor cell activates LET-60 in the underlying vulval precursor cells (VPCs) through the EGF receptor LET-23 . LET-60 is required for three VPCs, P5.p, P6.p, and P7.p, to adopt appropriate vulval fates and form a vulva composed of 22 cells (Shin & Reiner, 2018) . In contrast to excretory duct development, there were no defects in vulval induction and patterning in the L3 and L4 larval stages in mNG:: LET-60 animals. Notably, *let-60 ( n2021 )* animals had significant vulval induction defects ( **Figure 1C** ). This indicates that mNG:: LET-60 function is sufficient for VPC fate induction. Interestingly, early larval survival and vulval induction involve many of the same components in the RTK-Ras-ERK signaling pathway: LET-23 /EGFR > SEM-5 /Grb2 > SOS-1 > LET-60 /Ras > LIN-45 /Raf (Abdus-Saboor et al., 2011; Shin & Reiner, 2018) . This suggests that mNG:: LET-60 interferes with some other element of Ras regulation that is unique in the excretory duct. One possibility is LET-60 /Ras interaction with GAP-2 , which regulates LET-60 function in excretory duct development but not during vulval induction (Gyurko et al., 2015). Consistent with this idea, GAP proteins interact with the N-terminal region of Ras proteins (Hobbs et al., 2016) . Thus, the mNG fused to the N-terminus of LET-60 could interfere with GAP-2 interactions with LET-60 . Alternatively, there might be differences in LET-60 /Ras cellular regulators or tissue-specific post-translational modifications of LET-60 required in the excretory duct that are disrupted by mNG tagging (Fernandez-Medarde et al., 2021). Overall, the modest or absent impact on LET-60 /Ras function suggests that mNG:: LET-60 likely displays many normal aspects of LET-60 localization.

Examination of mNG:: LET-60 by confocal microscopy revealed widespread tissue expression in larvae and adults, consistent with a previous transcriptional reporter (Dent & Han, 1998) . Further, there were differences in levels of protein in cells and distinctions in subcellular localization. For example, mNG:: LET-60 was present at high levels in VPCs during VPC induction in the L3 larval stage ( **Figure 1D** ), where it localized predominantly to the intracellular region—cytosol and possibly endomembranes. We also observed that LET-60 levels were significantly higher in the 1° VPCs compared to the 2° and 3° VPCs during VPC induction where LET-60 /Ras activity is higher ( **Figure 1E** ) (Shin & Reiner, 2018) . Interestingly, mNG:: LET-60 Ras was present at low levels in the uterine anchor cell and during early stages of VPC induction was largely found in the anchor cell nucleus ( **Figure 1D** ). Although LET-60 is not yet known to have a function in the anchor cell, LET-23 /EGFR acts in the anchor cell to promote proper alignment with the vulval cells, where it could signal through LET-60 /Ras (Spiri et al., 2022) . Immunolocalization and biochemical isolation studies have revealed that vertebrate KRas and HRas localize to nuclei of several cell types (Contente et al., 2011; Fuentes-Calvo et al., 2010) , but whether Ras functions in the nucleus is unclear. High levels of mNG:: LET-60 were observed in the sex myoblast cells in the intracellular region and nucleus during the L3 and L4 larval stages ( **Figure 1F** ), where LET-60 /Ras regulates sex myoblast migration and differentiation (Sundaram et al., 1996; Sundaram, 2013) . High levels of mNG:: LET-60 was also observed in tissues where LET-60 /Ras is not known to function, such as nuclear and occasional nucleolar localization of mNG:: LET-60 in intestinal cells in early larval stages ( **Figure 1G** ), regionalized intracellular localization in the pharynx ( **Figure 1H** ), and high levels of intracellular mNG:: LET-60 accumulation in the spermatheca ( **Figure 1I** ). Enriched intracellular mNG:: LET-60 was detected in neurons associated with the nerve ring ( **Figure 1J** ), consistent with *let-60* mRNA expression and LET-60 function in olfactory neurons whose cell bodies and axons are located within the nerve ring (Hirotsu et al., 2000; Menini et al., 2010; Taylor et al., 2021) . Plasma membrane localization of mNG:: LET-60 was found throughout the germline, in body wall muscle cells, and hypodermal seam cells ( **Figure 1K -M** ), which are tissues where LET-60 /Ras has known functions (Sundaram, 2013) . We also noted high levels of intracellular and nuclear mNG:: LET-60 in the somatic sheath cells of the gonad ( **Figure 1K** ), where LET-60 might regulate germline morphogenesis (Lu et al., 2008) .

Our studies revealing distinct levels and subcellular localization of mNG:: LET-60 is consistent with the notion that the levels and compartmentalized localization of Ras can specify diverse Ras signaling activities through interactions with distinct pools of Ras activators and effectors (Kolch et al., 2023; Prior & Hancock, 2012) . An interesting observation from our investigation is the strong localization of mNG:: LET-60 to the intracellular cytosolic region of many cell types and the lack of clear plasma membrane or endomembrane localization where Ras proteins are thought to signal (Hernandez-Valladares et al., 2014) . Notably, subcellular fractionation studies in vertebrate MDCK cells have found that the majority of endogenous Ras is cytosolic (Choy et al., 1999) . This includes significant amounts of farnesylated Ras (Gutierrez et al., 1989) , suggesting that Ras might be maintained in the cytosol by a chaperone(s) that shields the lipid anchor and prevents localization to membranes (Wright & Philips, 2006) . It is possible that strong intracellular cytosolic mNG:: LET-60 localization obscures lower signaling levels of mNG:: LET-60 associated with cell membranes and internal endomembranes. Importantly, although the membrane localization was most prominent in the germ cells, the overall intracellular signal in germ cells was also high. A comparison with 1° VPCs, which lack clear membrane localization, indicated that the level (mean fluorescence intensity) of intracellular mNG:: LET-60 in the germ cells (13322 ± 1425 A.U.) was similar to the 1° VPCs intracellular signal (11760 ± 2016, n > 5 animals for each, **Methods** ). Thus, the absence of a clear membrane signal of mNG:: LET-60 in cells is not likely caused by a saturation effect ( *i.e.,* only a limited amount of LET-60 /Ras can associate with membranes). We suspect that mNG:: LET-60 localization is likely similar to untagged LET-60 , as strong intracellular mNG:: LET-60 accumulation occurs in the VPCs and mNG:: LET-60 signals normally here to induce VPC fate. Taken together, our observations suggest that mNG:: LET-60 can be a valuable reagent to examine LET-60 levels and localization in the cells and tissues where LET-60 functions. However, the function of mNG- LET-60 in the developmental or physiological process that LET-60 regulates should be evaluated to make certain that it is not perturbed, which will provide support that mNG:: LET-60 is localized normally.

## Methods

***C. elegans* culture:**

C. elegans were reared at 16°C, 18°C, or 20°C on nematode growth medium plates containing OP50 *Escherichia* coli according to standard procedures (Stiernagle, 2006) . Strains used in this study are described in the Strain Table.

**Generation of genome-edited *mNG:: let-60* strain:**

The N-terminal mNG knock-in allele *let-60 ( qy220 )* was created using CRISPR/Cas9 genome editing with a self-excising cassette for hygromycin selection as described previously (Dickinson et al., 2015; Keeley et al., 2020) . The sgRNA sequence directing Cas9 cleavage near the N-terminus is provided in Figure 1A . Note that a four amino acid linker (Ser-Thr-Lys-Glu) was incorporated in-frame between the mNG and the *let-60* coding sequence.

**Phenotypic analyses:**

To quantify L1 viability, between 20 to 40 embryos were singled to OP50 plates and incubated at 20°C. 24 h later, hatched viable larvae that had grown to the L2, or rod-like larvae that died in the L1 or early L2 were counted. We observed apparent embryonic lethality in a fraction of *let-60 ( n2021 )* and *let-60 ( qy220 )* embryos, and these were excluded from the analysis.

Vulval induction was scored as described previously (Moghal & Sternberg, 2003) . Briefly, vulval nuclei in well-fed L4 animals were examined to extrapolate how many of the P5.p, P6.p, and P7.p VPCs were induced to adopt vulval fates. In wild-type animals, each VPC gives rise to seven or eight great granddaughters (1.0 score for each fully induced VPC cell, for a total of 3.0). In conditions of incomplete VPC induction, the presence of three or four great granddaughters arising from a VPC was scored as 0.5, while less than three was scored as 0.

**Microscopy and image processing:**

All images were acquired on a Zeiss Axio Imager A1 microscope at 20°C controlled by the μManager software v.1.4.23 (Edelstein et al., 2010) equipped with a Hamamatsu ImagEM electron multiplying charge-coupled device camera, Zeiss 40× and 100× Plan Apochromat (1.4 numerical aperture) oil immersion objectives, Yokogawa CSU-10 spinning disc confocal scan head, and a 488 nm laser line. Worms were mounted on 5% noble agar pads with 0.01 M sodium azide and coverslipped for imaging.

All images shown are single z-slices where the tissues of interest were sharply in focus. Note that acquisition settings were optimized for each panel to clearly show the relevant tissues. To compare mNG:: LET-60 fluorescence intensity in the germ cells and VPCs, images were taken at the same magnification and acquisition settings. Images were processed in Fiji 2.0 (Schindelin et al., 2012) . We adjusted brightness and contrast to optimize the clarity of fluorescence images. The unsharp mask filter was applied to brightfield images to reduce blur.

**Fluorescence intensity quantification:**

All quantifications of mean fluorescence intensity were performed on raw images in Fiji 2.0. To measure mNG:: LET-60 fluorescence intensity in the 1°, 2°, and 3° VPCs, we drew ~ 30 x 20 µm boxes adjacent to VPC nuclei. We included all 2° VPCs (P5.p and P7.p) and 3° VPCs (P3.p, P4.p, and P8.p) in our analyses as we did not detect differences in signal levels within VPC groups at the P6.p 1-cell stage. To compare mNG:: LET-60 fluorescence intensity in the germ cell and 1° VPC intracellular regions, we drew ~ 5 x 5 µm boxes. Fluorescence intensity measurements were not background corrected as mNG:: LET-60 signal was ubiquitous in the germline and VPC fields of view and slide background during image acquisition did not vary.

**Statistical analysis:**

For comparisons of two categorical variables in the phenotypic analyses, we used the Fisher’s exact test. For comparisons of means between three populations in the LET-60 fluorescence intensity analysis, we performed the Kruskal-Wallis *H* test with a post hoc Dunn’s test as the datasets did not follow a Gaussian distribution as assessed by the D’Agostino-Pearson normality test. Sample sizes and *p* values are reported in the figure.

## Reagents

**Strains**

**Table:** 

**Strain**	**Genotype**	**Source**
N2	Wild-type (ancestral)	Caenorhabditis Genetics Center (CGC)
MT4866	*let-60 ( n2021 ) IV *	CGC
NK2987	*let-60 ( qy220 [mNG:: let-60 ]) IV *	This study
TU3310	*uIs59 [unc-119p::YFP] *	CGC
